# Hypoxia Enhances Glioma Resistance to Sulfasalazine-Induced Ferroptosis by Upregulating SLC7A11 via PI3K/AKT/HIF-1*α* Axis

**DOI:** 10.1155/2022/7862430

**Published:** 2022-11-18

**Authors:** Shicheng Sun, Changfa Guo, Taihong Gao, Dengzhen Ma, Xiangsheng Su, Qi Pang, Rui Zhang

**Affiliations:** Department of Neurosurgery, Shandong Provincial Hospital Affiliated to Shandong First Medical University, Jinan, 250021 Shandong, China

## Abstract

Glioma is the most common primary brain tumor, with a high rate of recurrence and treatment resistance. Glioblastoma is highly invasive, infiltrating surrounding brain parenchyma, and is known to cause intracranial metastasis resulting in a dismal prognosis. Hypoxia contributes significantly to chemo- and radiotherapy resistance in cancer. Ferroptosis is a nonapoptotic oxidative cell death that has been identified as a potential anticancer mechanism. Sulfasalazine (SAS) activates ferroptosis and plays a potential role in tumor treatment. However, the relationship between hypoxia and SAS resistance has not been elucidated. This study is aimed at investigating the role of hypoxia in SAS-induced ferroptosis and the underlying mechanisms. Here, we found that hypoxia significantly suppressed SAS-induced ferroptosis by upregulating SLC7A11 expression in the U87 and U251 glioma cell lines. Hypoxia promotes SLC7A11 expression by enhancing the PI3K/AKT/HIF-1*α* pathway. The AKT inhibitor MK-2206 and HIF-1*α* inhibitor PX-478 significantly reversed this effect. In addition, under normoxia, PX-478 induced a higher lipid peroxidation level by decreasing SLC7A11 expression in the U87 and U251 cells but could not induce cell death directly; it could significantly enhance the tumor cell killing effect of SAS. In vivo, the combination of PX-478 and SAS had a coordinated synergistic effect on anticancer activity, as revealed by subcutaneous and orthotopic xenograft mouse models. In conclusion, hypoxia enhanced glioma resistance to SAS-induced ferroptosis by upregulating SLC7A11 via activating the PI3K/AKT/HIF-1*α* axis. Combination therapy with PX-478 and SAS may be a potential strategy against glioma.

## 1. Background

Glioma is the most common primary malignant brain tumor in adults, glioblastoma is its most malignant and aggressive form, and they typically arise from glial or precursor cells and develop into astrocytoma, oligodendroglioma, ependymoma, or oligoastrocytoma [1]. Glioblastoma is an anaplastic, poorly differentiated malignant tumor with a peak incidence between 45 and 70 years [2]. Despite recent advances in diverse therapies for glioma, including surgery, radiotherapy, and chemotherapy, the median survival remains approximately at 15 months [3, 4]. Therefore, developing more efficient therapeutic strategies is imperative.

The tumor microenvironment (TME) refers to the local biological environment in which solid tumors are located [5]. The TME often displays at least some degree of hypoxia [6]. Coincidentally, gliomas undergo malignant progression under hypoxic conditions [7]. Tumor hypoxia is generated by irregular and tortuous vasculature formed within solid tumors, resulting in poor delivery of oxygen to cells. Hypoxia is associated with malignancy and tumor aggressiveness by increasing tumor cell proliferation and metastasis [8]. In addition, hypoxia has been associated with resistance to radiotherapy and chemotherapy. It also contributes to radioresistance by controlling several cellular processes, including cell cycle, apoptosis and senescence, creation of reactive oxygen species, invasion, and cancer cell stemness [9–12]. Previous studies showed that hypoxia also enhanced the resistance to temozolomide by different mechanisms, such as inducing ferritin light chain, regulating glioma stem cell properties, and mediating ATP-binding cassette proteins [13–15]. Hypoxia-inducible factor (HIF) is upregulated in the tumor hypoxia microenvironment, in which HIF-1*α* and HIF-2*α* are considered as the main response regulators [16]. Under hypoxia, HIF-1*α* is stabilized and translocated into the nucleus to play a role in transcription [17]. As a selective HIF-1*α* inhibitor, PX-478 interferes with HIF-1*α* and induces cell cycle arrest in cancer cells [18, 19]. Hypoxia and HIFs play important roles in glioma growth and survival through regulation of several key cell biological process, including glycolytic metabolism, angiogenesis, and drug resistance [20].

Ferroptosis is a nonapoptotic oxidative cell death that plays an essential role in various diseases, including cranial trauma, neuronal diseases, and brain tumors [21]. By analyzing 1,750 gliomas from four independent cohorts, ferroptosis was identified as the most enriched type of programmed cell death in glioma which was widely involved in malignancy progression and drug resistance [22]. Lipid peroxidation underlies the ferroptosis mechanism [23]. SLC7A11-glutathione peroxidase 4 (GPX4) axis, ferroptosis suppressor protein 1 (FSP1)-ubiquinol, and dihydroorotate dehydrogenase (DHODH)-ubiquinol axis constitute three major ferroptosis defense systems through the inhibition of lipid peroxidation. SLC7A11, the cystine/glutamate antiporter to synthesize GSH, neutralizes the oxidative substances in the cell membrane [24]. Sulfasalazine (SAS), a wildly recognized antirheumatoid arthritis drug, has been recently found to play an anticancer role in various cancers including malignant gliomas by activating ferroptosis by inhibiting SLC7A11 [25]. SAS had fewer side effects when applied in vivo and better water solubility than other ferroptosis inducers [26, 27]. To date, the relationship between hypoxia and SAS-induced ferroptosis in glioma has not been reported. In the present study, we investigated the role of hypoxia in SAS-induced ferroptosis in glioma cell lines and its underlying molecular mechanisms. Our study not only identified a novel mechanism for SAS resistance but also suggested a novel treatment through a combination of ferroptosis activator and HIF-1*α* inhibitor.

## 2. Material and Methods

### 2.1. Cell Lines

Human glioma cell lines U87 and U251 were directly purchased from Cell Bank of Type Culture Collection of Chinese Academy of Sciences (Shanghai, China). The U87 and U251 cell lines have been authenticated by STR profiling. These cells were cultured in high glucose Dulbecco's modified Eagle's medium (DMEM, Gibco, USA) with 10% fetal bovine serum (FBS, Biological, Industries) and 1% penicillin-streptomycin (10378016, Invitrogen, USA) at 37°C with 5% CO_2_.

### 2.2. Cell Growth and Cell Viability Assay

Cell growth was determined using a Cell Counting Kit-8 (CCK-8) assay kit (HY-K0301, MCE, Shanghai, China). The U87 and U251 cells were seeded into 96-well plates for 0, 24, 48, and 72 h at a density of 3000 cells per well. Then, 10 *μ*l CCK-8 solution was added to each well and incubated with the cells for 2 h. Absorbance (Abs) was detected at 450 nm using a microplate reader (Bio-Rad, Hercules, CA, USA).

For cell viability assay, the cells were seeded at 1000 cells per well in 96-well plates with fresh medium and analyzed by using the CCK-8 assay kit according to the above instructions. Reagent used contained SAS (HY-14655, MCE, USA), HIF-2*α*-IN-4 (HY-136748, MCE, USA), PX-478 (HY-10231, MCE, USA), and MK-2206 (HY-10358, MCE, USA). Cell viability = (Abs of the experimental group − Abs of the blank group)/(Abs of the control group − Abs of the blank group) × 100%. The half-maximal inhibitory concentrations (IC_50_) of SAS was determined using cell viability assay.

### 2.3. Vector Construction and Transduction

Stable overexpression of SLC7A11 and the control in cells were achieved by transfection with lentivirus synthesized by Genomeditech (Shanghai, China). The cells were infected with Lv-SLC7A11 or negative control lentivirus vectors. SLC7A11 siRNA was used to silence SLC7A11 gene expression. The target sequence of SLC7A11 was 5′-CCAUUAUCAUUGGCACCAUTT-3′. Scrambled siRNA targeting 5′TTCTCCGAACGTGTCACGT-3′ was used as a negative control.

### 2.4. RNA Extraction and Quantitative Real-Time PCR Array

Total RNA was extracted from cultured cells using a total RNA extraction kit (RC112-01, Vazyme Biotech, Nanjing, China). The concentration and purity of RNA were measured by the absorbance at 260 nm and the ratio of 260/280 nm in NanoDrop ND-1000 (NanoDrop, Wilmington, DE, USA). Total RNA from each sample was reversely transcribed using an all-in-one cDNA synthesis superMix (R333-01, Vazyme Biotech, Nanjing, China). SYBR Green PCR kit (R311-02, Vazyme Biotech, Nanjing, China) was used for real-time PCR. The primers of related genes are listed in Table S1. Quantitative PCR arrays are designed to analyze a panel of ferroptosis-related genes in human glioma cell lines U87 and U251 following the instructions of the manufacturer (Wcgene Biotechnology Corporation, China). The thermocycling conditions used in RT-qPCR were as follows: (1) 95°C for 30 s and (2) 95°C for 5 s and 60°C for 30 s (step 2 requires 45 cycles to be repeated).

### 2.5. Western Blot Analysis

Protein samples from the cells were lysed in cell lysis buffer (P0013C, Beyotime, Shanghai, China) containing protease/phosphate inhibitors (P1050, Beyotime Biotechnology, Shanghai, China). The concentration of the protein homogenates was determined using the BCA assay Kit (P0012, Beyotime, shanghai, China). Equal volumes of protein samples were separated by SDS-polyacrylamide gel electrophoresis and electrotransferred to PVDF membranes (Millipore, Billerica, MA, USA). And the percentage of polyacrylamide in SDS-PAGE is 10%. After being blocked with 5% nonfat milk dissolved in TBST (10 mM Tris, 150 mM NaCl, and 0.1% Tween-20; pH 7.6), for 2 h at room temperature, the membranes were incubated with the primary antibodies overnight at 4°C. Thereafter, membranes were incubated with the secondary antibodies coupled to horseradish peroxidase (HRP) for 1 h at room temperature. Protein bands were visualized with a supersensitive electrochemiluminescence (ECL) reagent (WBULS0100, Millipore, USA). The primary antibodies were goat polyclonal anti-SLC7A11/xCT (ab60171,ab175186, Abcam, USA, 1/1000), rabbit monoclonal anti-STEAP3 (ab151566, Abcam, USA, 1/1000), rabbit monoclonal anti-CA9 (ab108351, Abcam, USA, 1/500), rabbit polyclonal anti-*β*-actin (ab8227, Abcam, USA, 1/2000), rabbit monoclonal anti-HIF-1*α* (ab179483, Abcam, USA, 1/500), rabbit monoclonal anti-PI3K-gamma (ab32089, Abcam, USA, 1/1000), rabbit monoclonal anti-AKT (4691, CST, USA, 1/1000), and rabbit monoclonal anti-p-AKT (4060, CST, USA, 1/2000). The secondary antibody used in western blotting was goat anti-rabbit IgG H&L (HRP) (ab6721, Abcam, USA, 1/5000).

### 2.6. Immunofluorescence (IF) and Immunohistochemistry (IHC)

The U87 and U251 cells were grown on cover slides, fixed, blocked with 3% BSA, and permeabilized with PBS containing 0.1% *w*/*v* Triton X-100. The mouse brain of the orthotopic intracranial mouse model was cut in the thickness of 20 *μ*m by a frozen section and blocked with 3% BSA. For protein detection, the primary antibodies used have been already described. For IF, secondary antibodies were donkey anti-goat IgG H&L (ab150129, Abcam, USA). Nuclei were stained by 4,6-diamidino2-phenylindole (DAPI). Image acquisition was performed on ImageXpress Micro Confocal devices. IHC staining was performed by using Immunofluorescence Two-Step Test Kit (PV-9000, ZSGB-BIO, Beijing China) according to the manufacturer's instructions. Image acquisition was performed on an OLYMPUS BX63 microscope.

### 2.7. Clone Formation Assay

The U87 and U251 cells (3 × 10^2^ cells/plate) were seeded in 35 mm plates and grew for 2 weeks before being fixed with 4% paraformaldehyde for 15 min at room temperature. The cells were washed twice with PBS and stained with Crystal Violet Staining Solution (C0121, Beyotime Biotechnology, Shanghai, China).

### 2.8. TUNEL Assay

For the terminal deoxynucleotidyl transferase (TdT) dUTP nick-end labeling (TUNEL) assays, the U87 and U251 cells were seeded into 96-well plates at 1 × 10^4^ cells per well treated with saline, PX-478, SAS, or PX-478 combined with SAS for 24 hours. The cells were then stained with the TMR (red) TUNEL Cell Apoptosis Detection Kit (G1502, Servicebio, China) according to the manufacturer's protocol. Images were acquired with an ImageXpress Micro Confocal (Molecular Devices, USA), and the percentage of TUNEL-positive cells was calculated.

### 2.9. Cell Apoptosis Analysis by Flow Cytometry

Cell apoptosis was measured by the Annexin V-fluorescein isothiocyanate (FITC)/propidium iodide (PI) kit (BD556547, BD Bioscience, USA). Briefly, the U87 and U251 cells were seeded into 6-well plates treated with saline, PX-478, SAS, or PX-478 combined with SAS for 24 hours, and then, the cells were collected and washed twice with cold PBS. Next, the cells were resuspended in 1x binding buffer at a concentration of 1 × 10^6^ cells per ml and transferred 100 *μ*l of the solution (1 × 10^5^ cells) to a 5 ml culture tube. Incubate with 5 *μ*l Annexin V-FITC and 10 *μ*l PI for 15 min at (RT) 25°C in the dark, and then, add 400 *μ*l of 1x binding buffer to each tube. The results were analyzed using a FACS Calibur or an EPICS XL flow cytometer (BD Biosciences).

### 2.10. TCGA Data Analysis

mRNA expression data from the TCGA database was used to assess correlation between HIF-1*α* and SLC7A11 expressions. The analyses were performed using the online analysis software named cBioPortal (http://www.cbioportal.org). We used whole-exome and/or whole-genome sequencing of 257 tumor/normal pairs in glioma (TCGA, Cell 2013) study [28].

### 2.11. Malondialdehyde (MDA) Assay

The relative MDA concentration in cell lysate and tumor tissue was assessed using an MDA Assay kit (BC0025, Servicebio, China) according to the manufacturer's instructions [29]. Measure the absorbance at 532 nm using a microplate reader.

### 2.12. Subcutaneous Xenograft and Orthotopic Xenograft Mouse Model

All experimental animal procedures were conducted strictly by the Guide for the Care and Use of Laboratory Animals and approved by the Animal Care and Use Committee of the Shandong provincial hospital. The male BALB/c nude mice were randomized and divided into 5 groups in a blinded manner, each group including six 4-week-old nude mice. The animals were anesthetized by intraperitoneal injection of 0.6% pentobarbital sodium, 70 mg/kg, prior to intracranial injection. In the subcutaneous xenograft model, the 1 × 10^6^ U87 cells were subcutaneously implanted in the right flanks of nude mice. At the second week after injection, the control group was intraperitoneally administered with DMSO; meanwhile, orally give saline twice a day; SAS group was intraperitoneally administered with SAS (8 mg in 0.2 ml saline twice daily); meanwhile, orally give saline twice a day for two weeks; PX-478 group was intraperitoneally administered with DMSO; meanwhile, orally give PX-478 (100 mg/kg) twice a day; PX478+SAS group was intraperitoneally administered with SAS; meanwhile, orally give PX-478; PX-478+Lv-SLC7A11+SAS group was subcutaneously implanted 1 × 10^6^ Lv-SLC7A11-transfected U87 cells and was intraperitoneally administered with SAS; meanwhile, orally give PX-478. From the eighth day of the experiment, tumor size was monitored by measuring length (*L*) and width (*W*) with a Vernier caliper every 3 days. The volume of subcutaneous tumor was calculated using the following formula: *V* = *L* × *W*^2^/2. In addition, we comprehensively evaluated the state of experimental animals by observing their hair color, abdominal respiration, external genitalia, claw and toe characteristics, etc. At day 23 after implantation, subcutaneous tumors were collected, fixed with 4% paraformaldehyde, and sectioned for measurement. The largest subcutaneous tumor in mice was 9.6 mm in length, 8.7 mm in width, and 363.3 mm^3^ in volume.

In the orthotopic xenograft mouse model, each mouse was intracranially injected with 1 × 10^5^ luciferase-transfected U87 cells in 10 *μ*l PBS solution using a stereotactic head frame at a depth of 3 mm through a bur hole placed 2 mm lateral and 2 mm posterior to the bregma. At the second week after injection, mice received SAS and PX-478 treatment the same as subcutaneous xenograft model for 2 weeks. In vivo tumor growth was monitored with a Xenogen IVIS Spectrum system (PerkinElmer) every week after implantation.

Humane endpoints were established in our study are as follows: (1) significant weight loss for 4 consecutive days; (2) 20% less weight than before the study; (3) Inability to eat or drink; (4) dyspnea: typical symptoms are mouth and nose salivation and/or cyanosis; (5) persistent diarrhea, treatment ineffective; (6) organ failure, respiratory distress; and (7) animals are on the verge of death or unable to move, or do not respond to gentle stimulation. We euthanized the experimental animals by carbon dioxide inhalation asphyxiation.

### 2.13. Lipid Peroxide Assay

Lipid peroxide level was analyzed by flow cytometry using C11-BODIPY dye. The U87 and U251 cells were seeded into 6-well plates at 3 × 10^5^ cells per well and treated with ferroptosis inducer SAS as well as ferroptosis inhibitor ferrostatin-1 (HY-100579, MCE, Shanghai, China). The culture medium was replaced with 1 ml medium containing 10 *μ*M of C11-BODIPY (D3861, Thermo Fisher Scientific, USA), and then, the cells were incubated for 30 min in a humidified incubator (at 37°C, 5% CO2). The media then were removed and washed cells with PBS three times. For measurement using fluorescence microscope, images were acquired with an ImageXpress Micro Confocal (Molecular Devices, USA). For measurement using flow cytometry, the cells were harvested in 15 ml tubes and resuspended in 500 *μ*l of PBS. The cell suspension was filtered through cell strainer (0.4 *μ*m nylon mesh). The results were analyzed using a FACS Calibur or an EPICS XL flow cytometer (BD Biosciences).

### 2.14. Transmission Electron Microscopy Assay

Transmission electron microscopy analyses were conducted [30]. Briefly, the U87 and U251 cells were seeded into 6-well plates at 3 × 10^5^ cells per well and exposed to saline, PX-478, or Lv-SLC7A11 combined with PX-478 for 24 h. After that, the cells were collected, washed three times with PBS, and fixed with 2.5% glutaraldehyde. The samples were then pretreated according to standard procedures including staining, dehydration, embedding, and slicing to obtain ultrathin sections. During the analysis, images were acquired using a HITACHIH-7000 transmission electron microscope (TEM; Hitachi, Tokyo, Japan).

### 2.15. Statistical Analysis

Data were presented as mean values ± standard deviation (SD) from at least three experiments. Student's unpaired *t*-test used to analyze the differences between two groups and one-way analysis of variance (ANOVA) used for the comparison among three or more groups were conducted using GraphPad Prism 8 (GraphPad Software, CA, USA) and SPSS (IBM, NY, USA). We used Student-Newman-Keuls (SNK) *q* test (*n* < 4) and least significant difference (LSD) *t*-test (*n* > 4) to perform ANOVA post hoc test. The correlation analysis was conducted using TCGA database by cBioPortal (http://www.cbioportal.org/). Statistical analysis contained Spearman's correlation analysis and Pearson's correlation analysis. *P* values less than 0.05 were considered statistically significant (^∗^*P* < 0.05 and ^∗∗^*P* < 0.01).

## 3. Results

### 3.1. Hypoxia Protected Glioma Cells against SAS-Induced Ferroptosis

**Figure 1:**
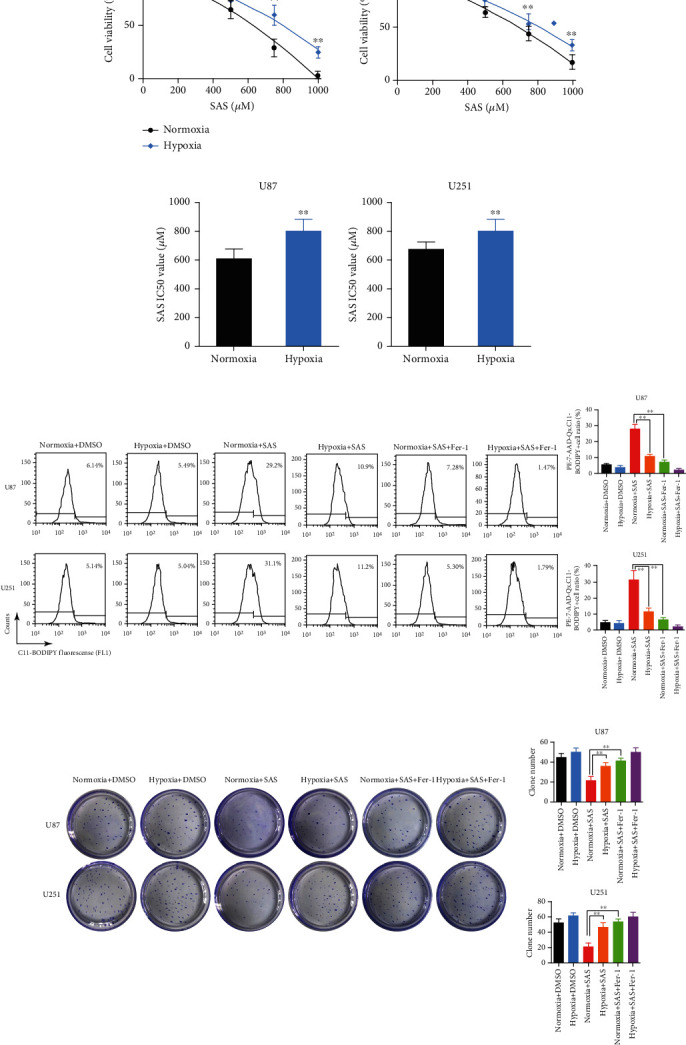
Hypoxia enhanced resistance of glioma cells to SAS-induced ferroptosis. (a) Cell viability curves at different concentrations of SAS for 24 h. Glioma cells under hypoxic environment became less sensitive to SAS. (b) Half-maximal inhibitory concentrations (IC_50_) were calculated in each group. Hypoxic cells had an obvious higher IC_50_ to SAS. (c) Lipid peroxidation assessed in the U87 and U251 cells after exposure to hypoxia, SAS, and Fer-1 by flow cytometry using C11-BODIPY. (d) Clone formation assay of cancer cells in the absence or presence of SAS or Fer-1 under normoxia or hypoxia. The bar graph showed mean ± SD of 3 independent experiments. ^∗^*P* < 0.05 and ^∗∗^*P* < 0.01.

Hypoxia (1% O_2_, 5% CO_2_, and 94% N_2_) is a crucial factor contributing to the aggressive behavior of gliomas [31]. We first explored cell viability at different concentrations of SAS for 24 h. We found that glioma cells in a hypoxic environment became less sensitive to SAS (Figure 1(a)). The half-maximal inhibitory concentrations (IC_50_) of the hypoxic U87 and U251 cells to SAS were significantly higher than that of normoxic cells (Figure 1(b)). Furthermore, the lipid peroxidation level was measured by flow immunolabeling using a C11-BODIPY probe. The results showed that the high lipid peroxidation level induced by SAS (500 *μ*M, 24 h) was decreased under hypoxia condition or by ferroptosis inhibitor Fer-1 (ferrostatin-1, 0.5 *μ*M, 24 h) incubation (Figure 1(c)). Moreover, we explored the effects of hypoxia and SAS on colony formation. We found that hypoxia and Fer-1 enhanced the colony-forming ability of cells treated with 500 *μ*M SAS for 2 weeks (Figure 1(d)). Together, these results suggest that hypoxic environment decreased the sensitivity of glioma cells to SAS.

### 3.2. SLC7A11 Acted as a Potential Effector Molecule of Hypoxia

**Figure 2:**
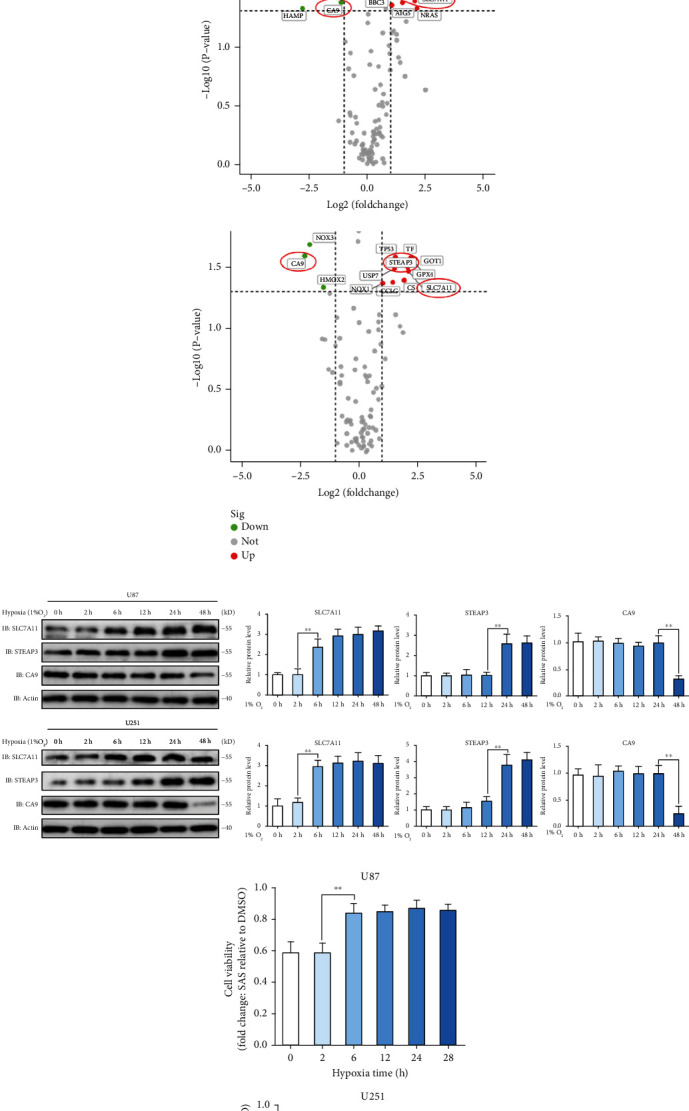
SLC7A11 was screened out as a potential effector molecule of hypoxia. (a) Ferroptosis-related qPCR array of normoxia and hypoxia identified several differentially expressed genes in the U87 and U251 cells. (b) The volcano map of differentially expressed genes depicted by difference degree and *P* value. SLC7A11, STEAP3, and CA9 were picked out. (c) Protein expression level of SLC7A11, STEAP3, and CA9 over a series of time points measured by western blot. SLC7A11 protein expression increased significantly at the sixth hour under hypoxia. (d) SAS resistance was measured by the ratio (cells exposed to SAS (500 *μ*M, 24 h)/cells exposed to DMSO). Glioma cells under hypoxic environment for at least 6 hours showed stronger SAS resistance. (e) Cell immunofluorescence showed that the fluorescence intensity of SLC7A11 protein in cells incubated under hypoxia for 6 hours was significantly higher than that in cells incubated under normoxia. The bar graph showed mean ± SD of 3 independent experiments. ^∗^*P* < 0.05 and ^∗∗^*P* < 0.01.

To identify potential genes that play vital roles in SAS resistance under hypoxic conditions, a ferroptosis profiling qPCR array was employed by comparing hypoxic cells and normoxic cells. Considering SAS would cause compensatory changes in many ferroptosis-related genes and ferroptosis profiling qPCR array limited the target genes to a small range, we choose to compare the gene changes between hypoxic and normoxic cells. Each group was repeated four times (Figure 2(a)). Heterogeneity of tumor cells resulted in diverse qPCR array results between the U87 and U251 cells. Considering that both cell lines showed decreased SAS sensitivity, we detected genes that changed significantly in both cell lines as potential targets. Three genes were identified as follows: SLC7A11, STEAP3 metalloreductase (STEAP3), and carbonic anhydrase 9 (CA9) (Figure 2(b)). Next, we determined the protein expression levels of the three genes over a series of time points by western blot. We found that the expression of SLC7A11 significantly increased at 6 h, the expression of STEAP3 significantly increased at 24 h, and the expression of CA9 significantly decreased between the 24th and 48th hours (Figure 2(c)).

Meanwhile, we explored the relationship between hypoxia and SAS resistance over a series of time points in the U87 and U251 cells. Durg resistance was measured by the ratio (cells exposed to SAS (500 *μ*M, 24 h)/cells exposed to DMSO). We found that hypoxia for 6 hours could induce a strong resistance to SAS (Figure 2(d)). Cell viability plot at the second hour and the sixth hour of hypoxic treatment in Figure S1A could clearly show that hypoxia for 6 hours could induce a strong resistance to SAS. The time course of SLC7A11 protein expression confirmed the trends of enhanced SAS resistance but not the time course of STEAP3 and CA9. To further verify SLC7A11 expression rule, we assessed SLC7A11 protein location and expression level via cellular immunofluorescence. The fluorescence intensity of SLC7A11 protein in cells incubated under hypoxia for 6 hours was significantly higher than that in cells incubated under normoxia, and the cell localization of SLC7A11 protein did not change significantly (Figure 2(e)). We conducted experiments to explore the SLC7A11 expression changes in patient samples (Figure S2A) and TCGA database (Figure S2B). Taken together, SLC7A11 acted as a potential effector molecule of hypoxia. Thus, we identified SLC7A11 as the target for further investigation.

### 3.3. Hypoxia Enhanced the Resistance to SAS via Increasing SLC7A11 Expression

**Figure 3:**
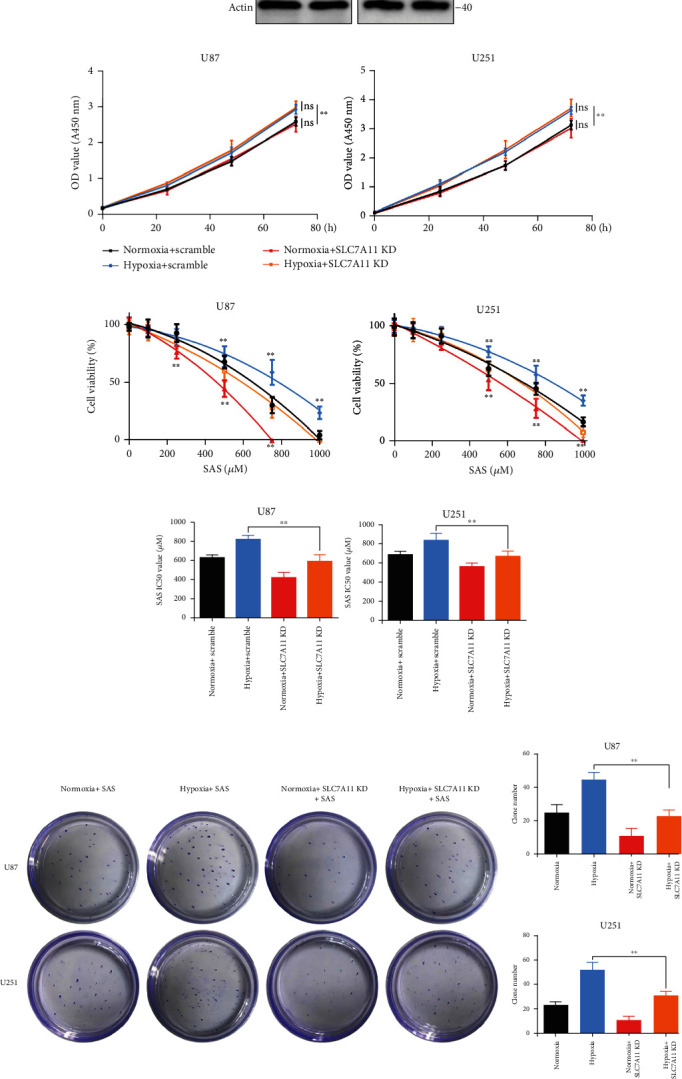
Hypoxia promoted SAS resistance via increasing SLC7A11 expression. (a) SLC7A11 protein expression level. SLC7A11 knockdown can significantly inhibit the protein expression level. (b) The growth curves were examined using a CCK-8 assay. SLC7A11 knockdown did not influence the cell proliferation rate of glioma cells. (c) Cell viability curves at different concentrations of SAS for 24 h. SLC7A11 knockdown could reverse the enhanced SAS resistance induced by hypoxia. (d) The IC_50_ of SAS in SLC7A11 knockdown group was obviously low than that of scramble group under hypoxic or normoxic condition. (e) Clone formation assay of cancer cells in the absence or presence of SAS for 2 weeks. SLC7A11 knockdown could reverse the enhanced cell colony formation ability induced by hypoxia. The bar graph showed mean ± SD of 3 independent experiments. ^∗^*P* < 0.05 and ^∗∗^*P* < 0.01.

To investigate the role of SLC7A11 in hypoxia-induced SAS resistance, we used SLC7A11 siRNA to inhibit SLC7A11 gene expression in the U87 and U251 cells. Firstly, we analyzed the efficacy of SLC7A11 knockdown (SLC7A11 KD). Western blot showed that siRNA significantly inhibited SLC7A11 protein expression (Figure 3(a)). The quantitative analysis results are shown in Figure S3. The CCK-8 cell viability assay showed that SLC7A11 knockdown could not influence the proliferation rate of glioma cells (Figure 3(b)). Cytotoxicity experiments showed that SLC7A11 knockdown could significantly reverse the enhanced SAS resistance of the U87 and U251 cells induced by hypoxia (Figure 3(c)). The IC_50_ of SLC7A11 knockdown cells was obviously lower than that of scramble cells under hypoxic conditions, which indicated that SLC7A11 knockdown could also reverse the increased IC_50_ of SAS induced by hypoxia (Figure 3(d)). Next, we explored whether SLC7A11 knockdown could influence SAS resistance in colony formation assay. We found that SLC7A11 knockdown reversed the enhanced cellular colony formation induced by hypoxia when treated with SAS (Figure 3(e)). In short, we identified SLC7A11 as a vital regulator gene for the ferroptosis defense ability of glioma cells under hypoxic conditions.

### 3.4. PI3K/AKT/HIF-1*α* Pathway Involved in the Upregulation of SLC7A11 Induced by Hypoxia

**Figure 4:**
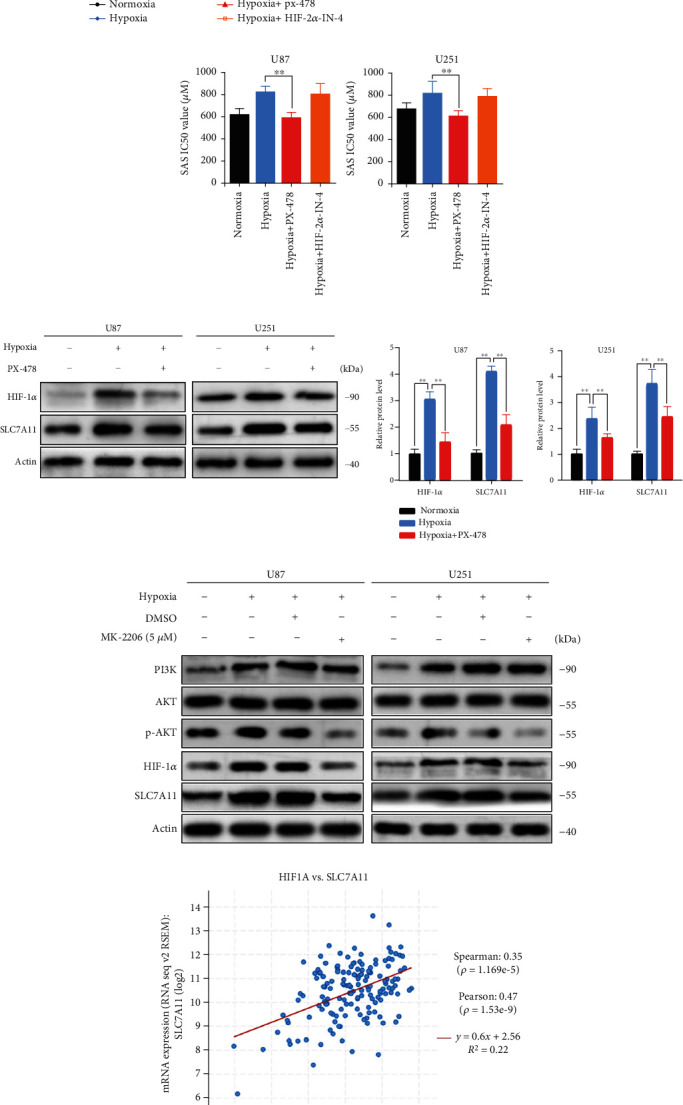
Hypoxia promoted SLC7A11 expression via PI3K/AKT/HIF-1*α* pathway. (a) Cell viability curves at different concentrations of SAS for 24 h. PX-478 can reverse the enhanced SAS resistance induced by hypoxia. (b) The IC_50_ of SAS in hypoxia with PX-478 group was obviously low than that of hypoxia group. The bar graph showed mean ± SD of 3 independent experiments. (c) Protein expression level of HIF-1*α* and SLC7A11 influenced by oxygen content and HIF-1*α* inhibitor PX-478 measured by western blot. PX-478 markedly decreased HIF-1*α* and SLC7A11 protein levels. (d) Protein expression level of PI3K, AKT, p-AKT, HIF-1*α*, and SLC7A11 influenced by oxygen content and AKT inhibitor MK-2206 measured by western blot. AKT inhibitor MK-2206 along with hypoxia attenuated the expression level of p-AKT, HIF-1*α*, and SLC7A11. (e) The relationship between HIF-1*α* and SLC7A11 expression level in TCGA. SLC7A11 was positively correlated with HIF-1*α* in 257 glioma tumor samples. ^∗^*P* < 0.05 and ^∗∗^*P* < 0.01.

Next, we would further explore how hypoxia induced SLC7A11 upregulation. Hypoxia-inducible factor (HIF) controlled a wide range of cell response to hypoxia, which included two major HIF-*α* subunits, HIF-1*α* and HIF-2*α* [32]. HIF-1*α* contributed more to the acute hypoxia-driven transcriptional responses [17], and some researchers had reported that HIF-1*α* was related to the SLC7A11 expression [33, 34]. To investigate the mechanism of SLC7A11 upregulation under hypoxia, we employed HIF-1*α* inhibitor PX-478 and HIF-2*α* inhibitor HIF-2*α*-IN-4. We found that treatment glioma cells with PX-478 under hypoxia could significantly reverse the enhanced ferroptosis resistance, but HIF-2*α*-IN-4 could not (Figures 4(a) and 4(b)). We explored the effects of PX-478 on the SLC7A11 expression. We found that PX-478 markedly decreased HIF-1*α* and SLC7A11 protein levels (Figure 4(c)). The PI3K/AKT pathway was reported to be the upstream of HIF-1*α* [35], which had been reported to participate in the malignant progression of glioma and was considered as an important regulator of ferroptosis [36, 37]. Therefore, we hypothesized that the increased SLC7A11 expression might be due to the activation of the PI3K/AKT/HIF-1*α* pathway. To prove our hypothesis, we explored the protein changes in the PI3K/AKT/HIF-1*α* pathway by western blotting. The results indicated that hypoxia activated PI3K and phosphorylated AKT, HIF-1*α*, and SLC7A11 expressions. Treatment with the AKT inhibitor, MK-2206, significantly attenuated the expressions of p-AKT, HIF-1*α*, and SLC7A11 (Figure 4(d)). The quantitative analysis results are shown in Figure S4. We then analyzed the public data and found that the expression of SLC7A11 was positively correlated with HIF-1*α* in 257 glioma tumor samples [28] (Figure 4(e)).

### 3.5. HIF-1*α* Inhibitor PX-478 Induced Lipid Peroxidation in Glioma Cells

**Figure 5:**
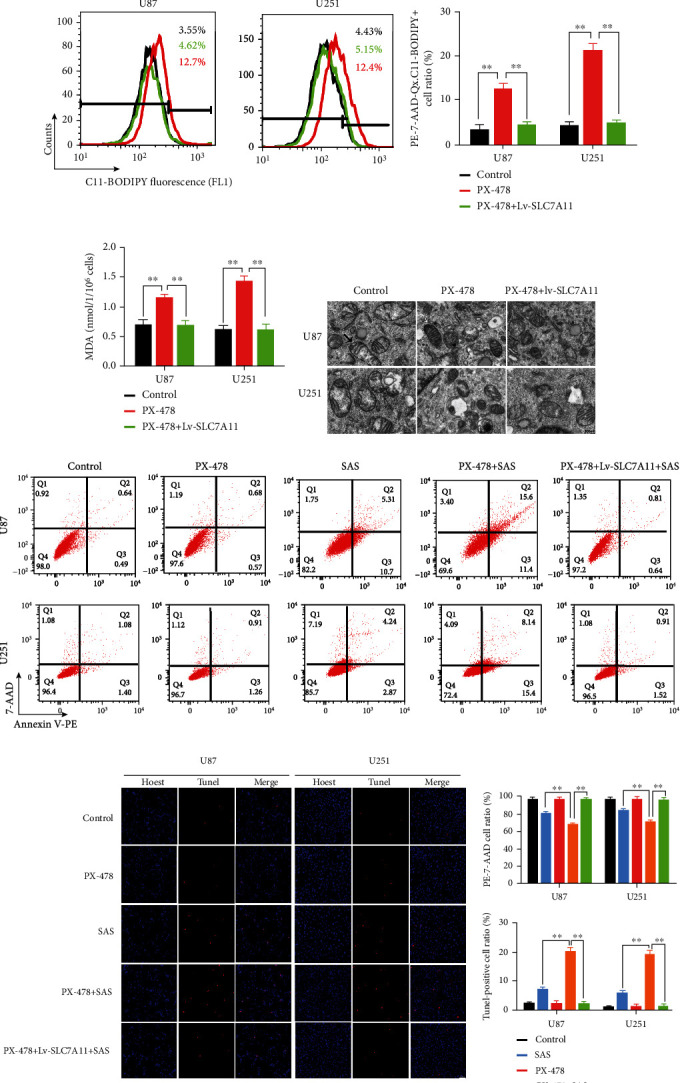
PX-478 induced lipid peroxidation in the U87 and U251 cells. (a, b) Lipid peroxidation assessment in U87 and U251 cells after exposure to DMSO, PX-478, or PX-478+Lv-SLC7A11 measured by fluorescence microscope (a) and flow cytometry (b). (c) Quantitative analysis of the expression levels of MDA in the U87 and U251 cells after exposure to DMSO, PX-478, or PX-478+Lv-SLC7A11. MDA increased after treating with PX-478 and reversed in the presence of Lv-SLC7A11. (d) Transmission electron microscopy images of the U87 and U251 cells after exposure to DMSO, PX-478, or PX-478+Lv-SLC7A11. PX-478-treated cancer cells exhibited shrunken mitochondria with enhanced membrane density. Black arrow: normal mitochondria; white arrow: shrunken and high membrane density mitochondria. Quantitative analysis of mitochondrial length was listed on the right. (e, f) Cell apoptosis was detected by flow cytometry (e) and TUNEL fluorescence staining (f) in the U87 and U251 cells after exposure to DMSO, PX-478, SAS, PX-478+SAS, and PX-478+Lv-SLC7A11+SAS. The bar graph showed mean ± SD of 3 independent experiments. ^∗^*P* < 0.05 and ^∗∗^*P* < 0.01.

Ferroptosis resistance can be induced by hypoxia; lipid peroxidation underlies the mechanism of ferroptosis [23]. We were determined to explore whether suppression of HIF-1*α* could directly induce lipid peroxidation in glioma cells. We utilized the C11-BODIPY probe and observed that the HIF-1*α* inhibitor, PX-478 (20 *μ*M), significantly increased lipid peroxidation levels in glioma cells. To determine whether SLC7A11, as a downstream effector of HIF-1*α*, could reverse the lipid peroxidation induced by PX-478, glioma cells were transfected with lentivirus that overexpresses human SLC7A11 cDNA (PX-478+Lv-SLC7A11 group). As expected, lipid peroxidation levels decreased in the PX-478+Lv-SLC7A11 group (Figure 5(a)). As depicted in Figure 5(b), the ratios of C11-BODIPY+ cells in the PX-478 treatment group were 12.7% and 21.4% in the U87 and U251 cell lines, respectively; those in the control group were 3.55% and 4.43%, respectively, thereby indicating that the lipid peroxidation level after treatment with PX-478 was significantly increased. Importantly, the combination of PX-478 and Lv-SLC7A11 resulted in a low lipid peroxidation level, with C11-BODIPY+ cell ratios of 4.62% and 5.15% in the U87 and U251 cells, respectively. Furthermore, we confirmed this by measuring malondialdehyde (MDA), a product of lipid-oxidized damage. The results showed that the lipid-oxidized damage level was increased after treatment with PX-478 and reversed in the presence of Lv-SLC7A11 (Figure 5(c)). Transmission electron microscopy revealed that PX-478-treated cancer cells exhibited shrunken mitochondria with enhanced membrane density, which are morphologic features of ferroptosis. The black arrays represent normal mitochondria. White arrays represent shrunken and high membrane density mitochondria. SLC7A11 overexpression reversed this effect (Figure 5(d)). We then studied the potential role of PX-478- in SAS-induced ferroptosis under normoxic condition. We found that treatment with PX-478 alone could not cause cell death, which suggested that a modest degree of lipid peroxidation induced by PX-478 did not directly lead to cell death. We examined the combined effects of SAS and PX-478. The results indicated that the combined medication had a significantly higher cell death rate than SAS alone, as revealed by flow cytometry analysis and TUNEL staining, while SLC7A11 overexpression blocked the cytotoxic effect (Figures 5(e) and 5(f)).

### 3.6. PX-478 Enhanced the Anticancer Activity of SAS In Vivo

**Figure 6:**
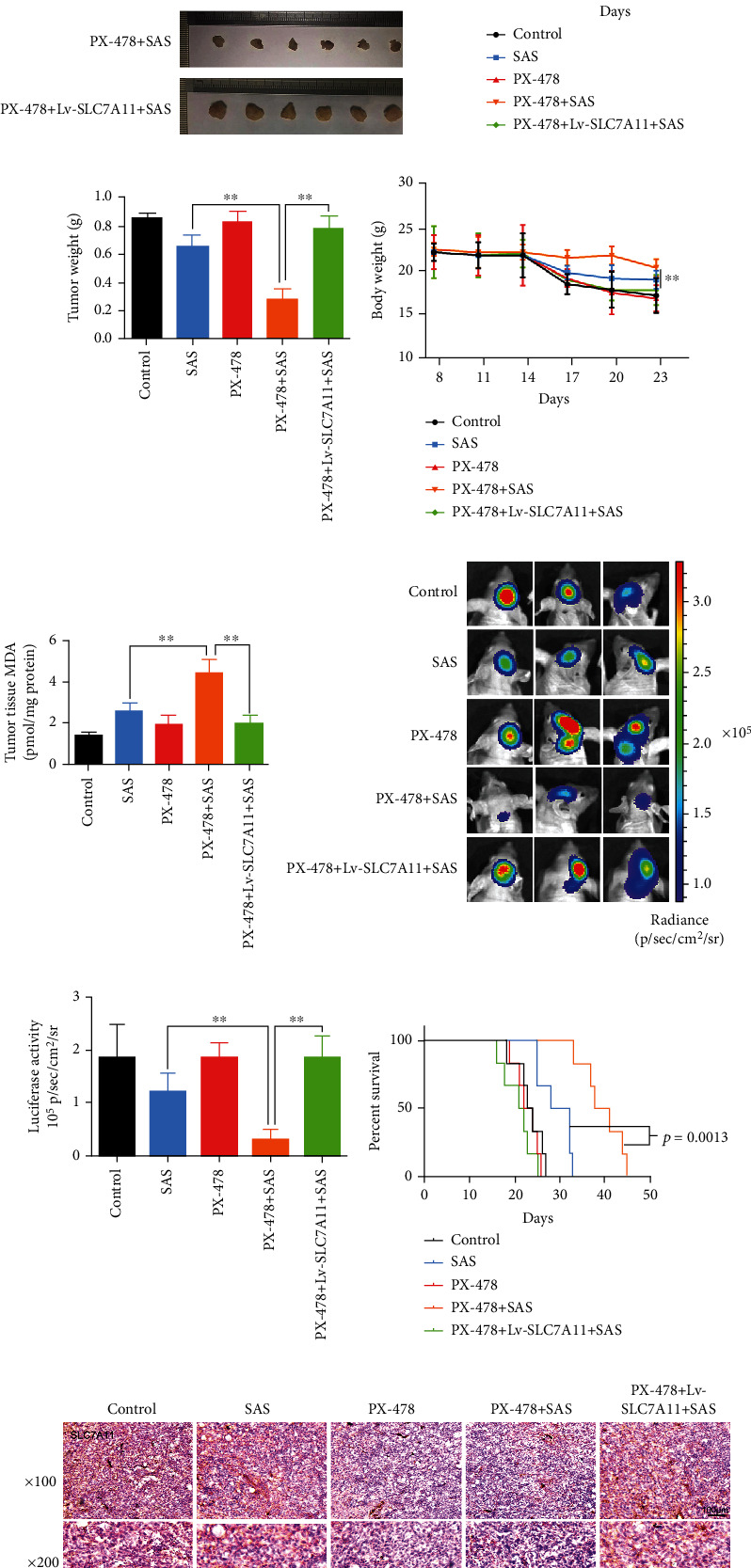
PX-478 promoted the anticancer activity of SAS in vivo. (a) Xenografts derived from the U87 or U87/Lv-SLC7A11 cells which were treated with different drugs demonstrated tumor volume (b), tumor weight (c), body weight (d), and tumor tissue MDA (e). (f, g) Luciferase activities verified the decreased and increased tumor growth, respectively, in orthotopic glioma models established by the U87 or U87/Lv-SLC7A11 cells. (h) Survival curves showed the survival rates of the engrafted mice. (i) Immunohistochemical labelling showed that the downregulated expression of SLC7A11 after being treated with PX-478 and Lv-SLC7A11 could efficiently increase SLC7A11 expression in vivo. The bar graph showed mean ± SD of 3 independent experiments. ^∗^*P* < 0.05 and ^∗∗^*P* < 0.01.

To further investigate the role of PX-478 in promoting SAS sensitivity *in vivo*, we established subcutaneous and orthotopic xenograft mouse models of glioma. In both models, SAS (8 mg in 0.2 ml saline twice daily) was intraperitoneally injected twice a day and PX-478 (100 mg/kg) was orally administered twice a day for 2 weeks. The detailed experimental process is presented in Material and Methods. Compared with the control group (intraperitoneal injection DMSO and oral given saline), the SAS group (intraperitoneal injection SAS and oral given saline) exhibited a certain degree of anticancer effect, but not the PX-478 group (intraperitoneal injection DMSO and oral given PX-478). The combination group (intraperitoneal injection SAS and oral given PX-478) had significantly better anticancer activity than the SAS group. However, the Lv-SLC7A11 group (implant with SLC7A11 overexpression cells, intraperitoneal injection SAS, and oral given PX-478) showed no anticancer effect, as measured by tumor volume, tumor weight, mice body weight, and tumor tissue MDA in the subcutaneous xenograft model (Figures 6(a)–6(e)). Similar results were obtained in the orthotopic xenograft model, as measured by luciferase activity (Figures 6(f) and 6(g)). The overall survival of the combination group was also higher than that of the SAS group (Figure 6(h)). Furthermore, histological analysis indicated that the PX-478 and combination groups exhibited weaker immunoreactivity for SLC7A11; Lv-SLC7A11 could efficiently increase SLC7A11 expression (Figure 6(i)). Collectively, PX-478 had very little therapeutic effect on glioma; however, it greatly enhanced the anticancer effect of SAS in both subcutaneous and orthotopic xenograft mouse models.

## 4. Discussion

The global incidence of glioma continues to increase along with the aging of the world's population; glioblastoma remains at a very low 5-year overall survival rate (6.8%) [38]. Tumor cells can develop resistance to various drugs during the therapy process, including ferroptosis-based drugs [39, 40]. In recent years, therapies underlying ferroptosis have attracted much attention with high expectations [41]. Hypoxia is correlated to the drug resistance of solid tumors. Alleviation of hypoxia is expected to sensitize the ferroptosis inducers toward solid tumors [42]. This study shows that hypoxia could enhance glioma resistance to SAS-induced ferroptosis. Therefore, a better understanding of the mechanism underlying SAS resistance may benefit ferroptosis-targeted treatment. We found that one of the most important underlying mechanisms of SAS resistance at the hyperacute phase of hypoxia is the upregulation of SLC7A11. SLC7A11, STEAP3, and CA9 are common genes that have changed significantly in both the U87 and U251 cell lines by PCR array. CA9 and STEAP3 have been reported to be important regulators of ferroptosis under hypoxia [43, 44]. CA9 has an intimate association with hypoxia and redox regulation [43]. Through western blot detection, we found that CA9 did not show its function in the hyperacute phase of hypoxia. We speculate that it may play a role in the chronic phase of hypoxia, which is also of great interest to us. STEAP3 increases sensitively to ferroptosis by regulating the p53/SLC7A11 pathway [44]. Namely, SLC7A11 played a central role in the hyperacute phase of hypoxia at least in the U87 and U251 cells, while CA9 and STEAP3 did not; and SLC7A11 was expressed stably in different types of cells and was considered as the most crucial gene at the hypoxia hyperacute phase.

The PI3K/AKT signaling pathway plays a central role in cell survival [37]. The allosteric AKT inhibitor, MK2206, has been reported to decrease tumor growth and enhance the antitumor efficacy of chemotherapeutic agents [45, 46]. We found that hypoxic activation of the PI3K/AKT/HIF-1*α* axis was essential for the upregulation of SLC7A11. Both the AKT inhibitor, MK-2206, and HIF-1*α* inhibitor PX-478, significantly inhibited SLC7A11 expression under hypoxic conditions. These results suggested that the upregulation of SLC7A11 induced by hypoxia was dependent on the activation of the PI3K/AKT/HIF-1*α* axis.

Clinical studies have demonstrated that the tumor hypoxic microenvironment is associated with poor prognosis in patients. Especially in advanced metastatic cancer, a hypoxic environment is often established, which plays an important role in cancer evolution [5]. In a hypoxic environment, activated HIF-1*α* promotes cancer migration in multiple ways, such as promoting EMT-related signaling, regulating the alignment of collagen fibers, and mediating the leakage and compression of blood and lymphatic vessels [47–49]. In recent years, the contribution of hypoxia to tumor therapy especially specific targeting of HIFs has been observed in a wide range of neoplastic cells [5]. In this study, we found new evidence about the relationship between hypoxia and ferroptosis, which is a potential therapeutic target in cancer therapy. Our research concluded that hypoxia-regulated SLC7A11 expression through PI3K/AKT pathway may provide a theoretical basis for clinical trials to help improve treatment outcomes. PX-478 had an antitumor effect with fewer side effects; it could penetrate the blood-brain barrier; however, its sole effect was limited [50]. As a result, scientists have paid much attention to its synergetic effect, including its combination with radiotherapy or immunotherapy [51, 52]. Radiotherapy and immunotherapy are related to ferroptosis [53, 54]. Given that HIF-1*α* regulates the ferroptosis core gene, SLC7A11, we hypothesized that PX-478 played a vital role in promoting ferroptosis by inhibiting SLC7A11 expression. We found that cells treated with PX-478 exhibited higher lipid peroxidation levels following C11-BODIPY staining. We also found deeply stained and shrunken mitochondria on transmission electron microscopy. The TME is characterized by regional hypoxia [55]. Therefore, we hypothesized that regional hypoxia in the TME in vivo would strengthen HIF-1*α*/SLC7A11 axis-regulated SAS resistance. We used subcutaneous and orthotopic xenograft mouse models to test the function of PX-478 in SAS therapy. Ideal experimental results were obtained; data showed that PX-478 can effectively decrease SLC7A11 expression in tumor tissue and promote the anticancer effect of SAS. With respect to how HIF-1*α* regulated SLC7A11, we performed ChIP with an anti-HIF-1*α* antibody and designed three pairs of primer to test whether HIF-1*α* can bind to the SLC7A11 promoter but did not obtain positive result. So, the regulatory mechanism of HIF-1*α* on SLC7A11 may be not direct transcriptional regulation, which may be a complicated process. Some researchers found HIF-1*α*/SLC7A11 pathway affected liver fibrosis through the mechanism of ferroptosis, but they did not explore the regulatory mechanism [34]. Another team of researchers found that HIF-1*α* improved the stability of SLC7A11 mRNA through lncRNA-PMAN, which may be an important mechanism of HIF-1*α* regulating SLC7A11 [33]. There may be other regulatory mechanisms which need further research.

**Figure 7:**
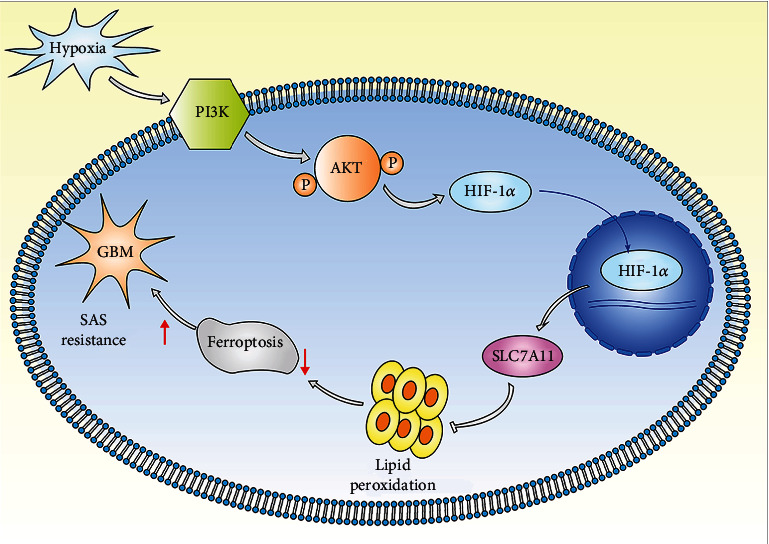
Pattern diagram of hypoxia modulation of ferroptosis. Hypoxia activated PI3K/AKT/HIF-1*α* pathway to increase SLC7A11 expression in the U87 and U251 cells. SLC7A11 protein neutralized lipid peroxidation to defend SAS-induced ferroptosis.

To the best of our knowledge, we first found that PX-478 could induce increased lipid peroxidation level and promote the anticancer effect of SAS in glioma. As for the mechanism, many factors could influence the activation and translocation of HIF-1*α* including growth factors, ROS, and various protein signaling pathways, which suggested that HIF-1*α* played vital roles not only under hypoxia [56]. HIF-1*α* also regulated SLC7A11 expression under normoxia [33, 34]. We found that the phenotype could be blocked by SLC7A11 overexpression, which indicated that the key effector under normoxia was SLC7A11. Additionally, this is the first study to uncover the relationship between the PI3K/AKT/HIF-1*α* pathway and ferroptosis. Beside PI3K/AKT/HIF-1*α* pathway, many other pathway also contributed to SLC7A11 expression. However, the pathways related to SLC7A11 expression were not related to hypoxia induction directly in glioma [57]. We first suggested that the combination of SAS and PX-478 had a good anticancer effect. Shortcoming of this study was that we did not test for other ferroptosis-related therapies and other HIF-1*α* inhibitory methods. In conclusion, we demonstrated that glioma resistance to SAS-induced ferroptosis was enhanced by hypoxia, owing to the activation of the PI3K/AKT/HIF-1*α* pathway and promotion SLC7A11 expression (Figure 7). A preprint has previously been published [58]. We demonstrated that PX-478 and SAS had a coordinated synergistic effect on anticancer activity, both in vitro and in vivo. Future studies should include other kinds of ferroptosis-related therapies to evaluate whether PX-478 not only promotes SAS action but also those of others that are sufficiently effective and safe for the treatment of human tumors.

## Data Availability

The datasets used and analyzed during the current study are available from the corresponding author on reasonable request.

## Abbreviations

TME: Tumor microenvironment
SLC7A11: Recombinant solute carrier family 7, member 11
HIF: Hypoxia-inducible factor
DMEM: Dulbecco's modified Eagle's medium
FBS: Fetal bovine serum
CCK-8: Cell Counting Kit-8
IC_50_: Half-maximal inhibitory concentration assay
ECL: Electrochemiluminescence
IF: Immunofluorescence
IHC: Immunohistochemistry
MDA: Malondialdehyde
STEAP3: STEAP3 metalloreductase
CA9: Carbonic anhydrase 9.

## Supplementary Materials

Supplementary Materials Table S1: primers used in this study. Figure S1: cell viability plot at the second hour and the sixth hour of hypoxic treatment. Figure S2: the SLC7A11 expression changes in patient samples and TCGA database. Figure S3: quantitative analysis of western blot in Figure 3(a). Figure S4: quantitative analysis of western blot in Figure 4(d).
