# Supplementary material for: Hypoxia Enhances Glioma Resistance to Sulfasalazine-Induced Ferroptosis by Upregulating SLC7A11 via PI3K/AKT/HIF-1α Axis
Source: Oxid Med Cell Longev. 2022 Nov 18;2022:7862430. doi: 10.1155/2022/7862430 (PMC9699746; doi:10.1155/2022/7862430)
Supplement: Supplementary Materials — Table S1: primers used in this study. Figure S1: cell viability plot at the second hour and the sixth hour of hypoxic treatment. Figure S2: the SLC7A11 expression changes in patient samples and TCGA database. Figure S3: quantitative analysis of western blot in Figure 3(a). Figure S4: quantitative analysis of western blot in Figure 4(d). [file 7862430.f1.zip › Supplementary information.docx]

**Supplementary information**

Hypoxia enhances glioma resistance to sulfasalazine-induced ferroptosis by upregulating SLC7A11 via PI3K/AKT/HIF-1α axis

Sun et al

**Contents**

Table S1. Primers used in this study

Figure S1. Cell viability plot at the second hour and the sixth hour of hypoxic treatment.

Figure S2. The SLC7A11 expression changes in patient samples and TCGA database.

Figure S3. Quantitative analysis of western blot in figure 3A.

Figure S4. Quantitative analysis of western blot in figure 4D.

Table S1: Primers used in this study

| **Genes** | **Sequence** |
| --- | --- |
| SLC7A11  (Human) | Forward: 5’- CTTTGTTGCCCTCTCCTGCTTC -3’  Reverse: 5’- CAGAGGAGTGTGCTTGTGGACA -3’ |
| HIF-1α  (Human) | Forward: 5’- TGCTTTAACTTTGCTGGCCC -3’  Reverse: 5’- GTTTCTGTGTCGTTGCTGCC -3’ |
| STEAP3  (Human) | Forward: 5’- CCCGTCCATTGCTAATTCCCT -3’  Reverse: 5’- CAGAAA AGAGACCCGAACCCA -3’ |
| CA9  (Human) | Forward: 5’- GGGTGTCATCTGGACTGTGTT -3’  Reverse: 5’- CTTCTGTGCTGCCTTCTCATC -3’ |
| β-Actin  (Human) | Forward: 5’- CACCATTGGCAATGAGCGGTTC -3’  Reverse: 5’- AGGTCTTTGCGGATGTCCACGT -3’ |

**
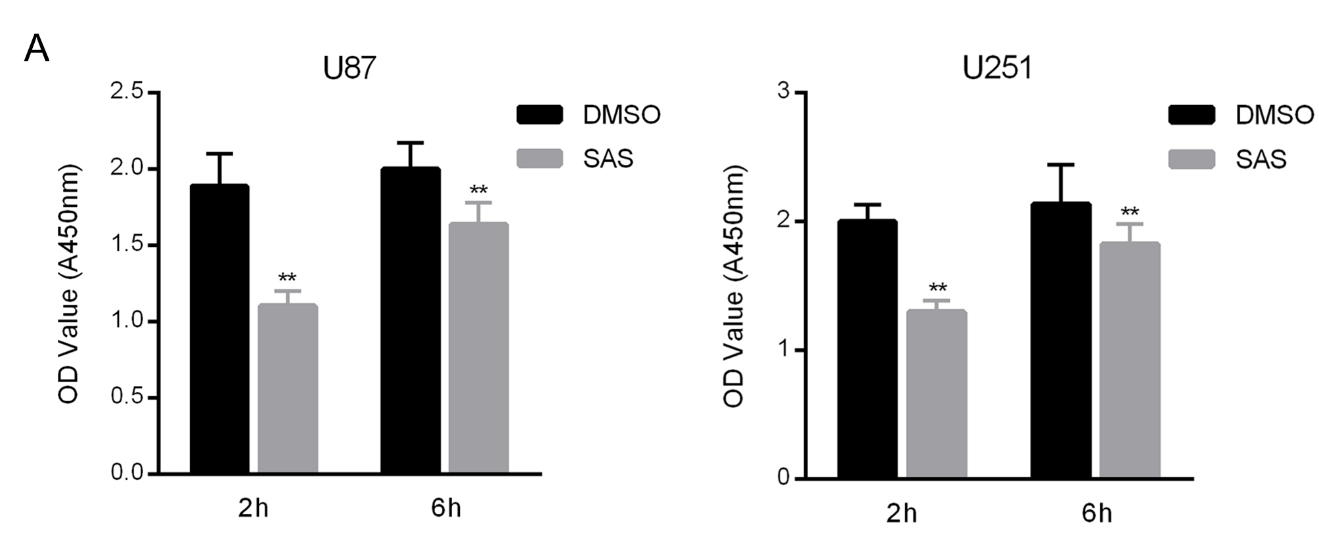
**

Figure S1. Cell viability plot at the second hour and the sixth hour of hypoxic treatment. The bar graph showed mean ± SD of 3 independent experiments. *P < 0.05, **P < 0.01.


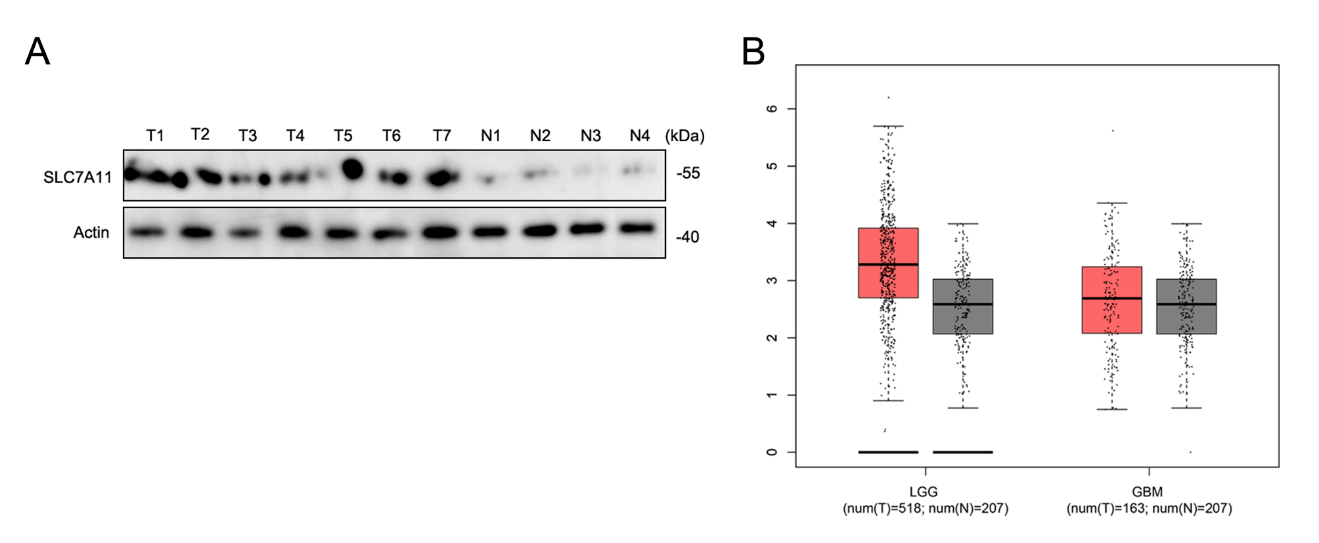


Figure S2. The SLC7A11 expression changes in patient samples and TCGA database.





Figure S3. Quantitative analysis of western blot in figure 3A. The bar graph showed mean ± SD of 3 independent experiments. *P < 0.05, **P < 0.01.


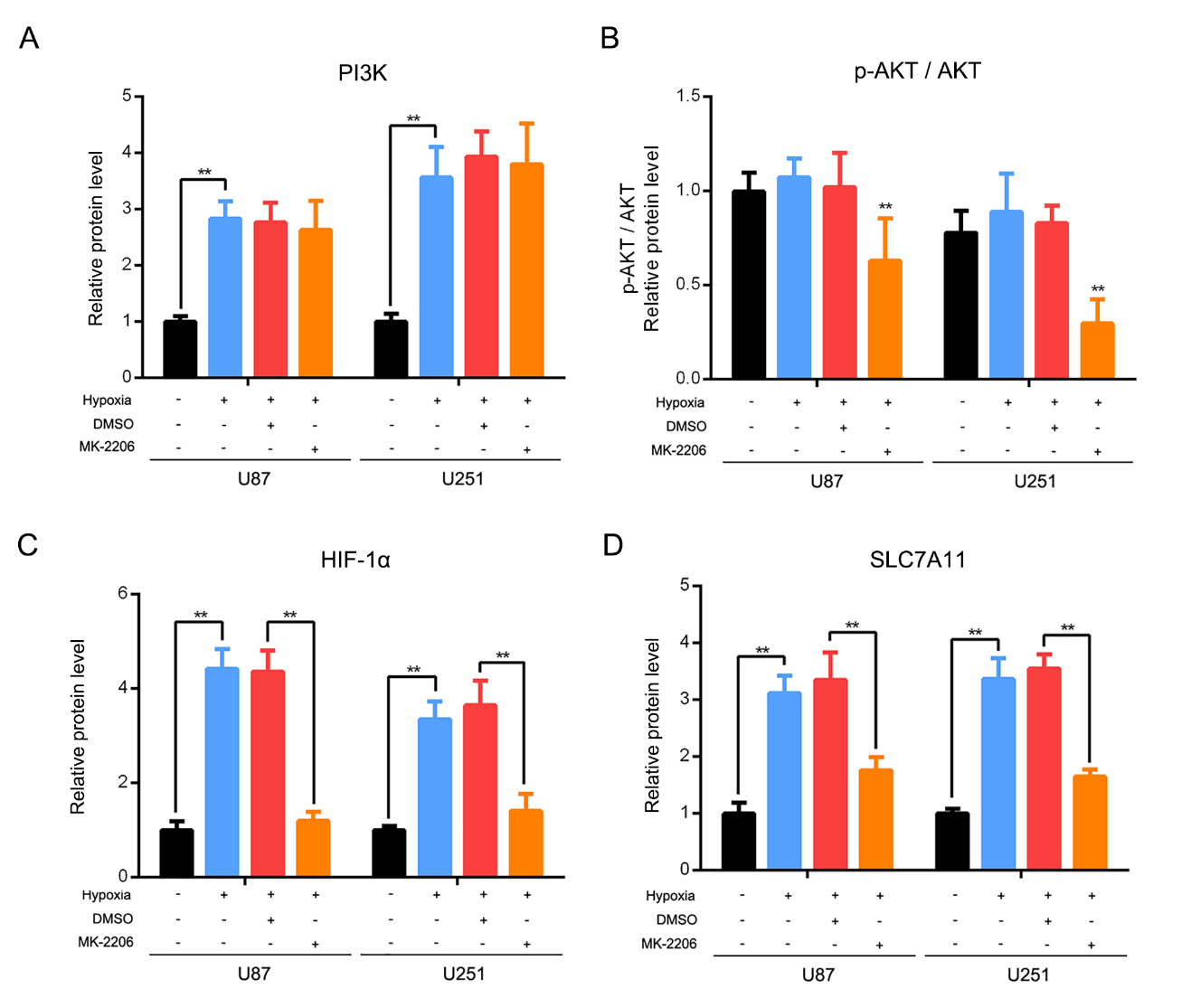


Figure S4. Quantitative analysis of western blot in figure 4D

A. Quantitative analysis of PI3K protein level. B. Quantitative analysis of AKT protein level. C. Quantitative analysis of p-AKT protein level. D. Quantitative analysis of HIF-1α protein level. E. Quantitative analysis of SLC7A11 protein level. The bar graph showed mean ± SD of 3 independent experiments. *P < 0.05, **P < 0.01.
