# Supplementary figures and images for: Hypoxia Enhances Glioma Resistance to Sulfasalazine-Induced Ferroptosis by Upregulating SLC7A11 via PI3K/AKT/HIF-1α Axis
Source: Oxid Med Cell Longev. 2022 Nov 18;2022:7862430. doi: 10.1155/2022/7862430 (PMC9699746; doi:10.1155/2022/7862430)

A

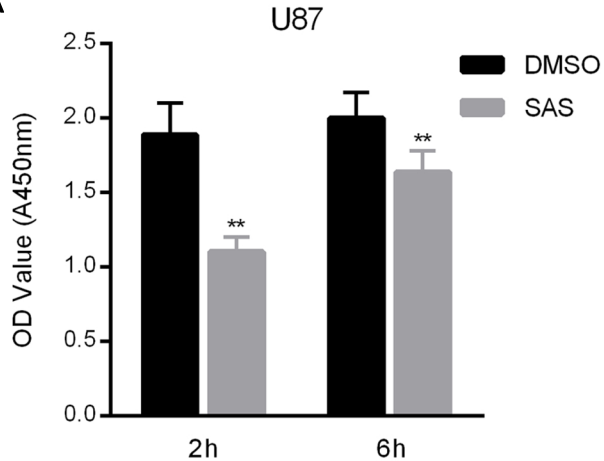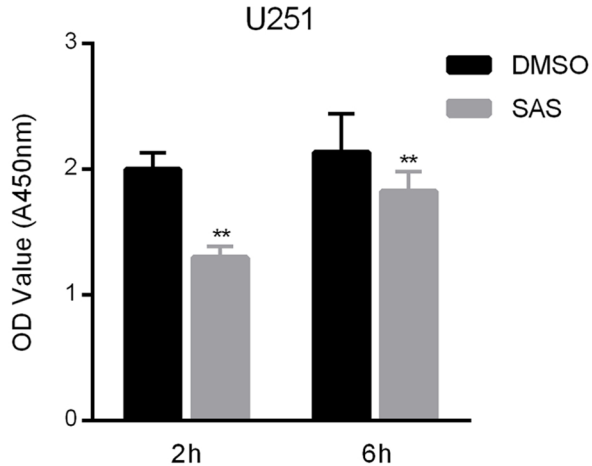

Supplement: Supplementary Materials — Table S1: primers used in this study. Figure S1: cell viability plot at the second hour and the sixth hour of hypoxic treatment. Figure S2: the SLC7A11 expression changes in patient samples and TCGA database. Figure S3: quantitative analysis of western blot in Figure 3(a). Figure S4: quantitative analysis of western blot in Figure 4(d). [file 7862430.f1.zip › Figure S1 (1).pdf]

A

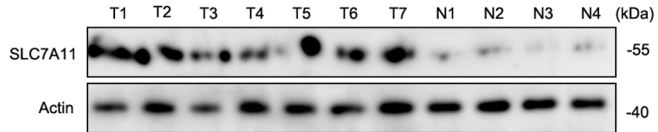

B

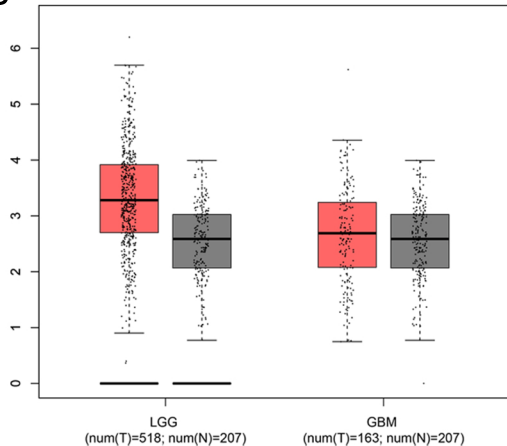

Supplement: Supplementary Materials — Table S1: primers used in this study. Figure S1: cell viability plot at the second hour and the sixth hour of hypoxic treatment. Figure S2: the SLC7A11 expression changes in patient samples and TCGA database. Figure S3: quantitative analysis of western blot in Figure 3(a). Figure S4: quantitative analysis of western blot in Figure 4(d). [file 7862430.f1.zip › Figure S2 (1).pdf]

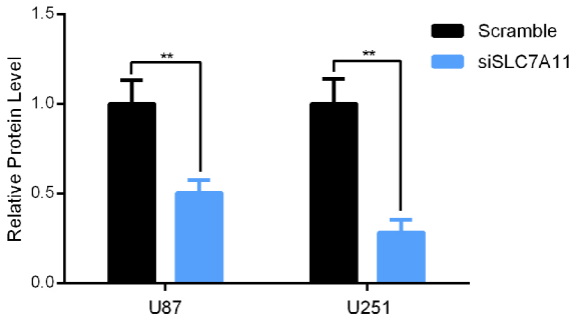

Supplement: Supplementary Materials — Table S1: primers used in this study. Figure S1: cell viability plot at the second hour and the sixth hour of hypoxic treatment. Figure S2: the SLC7A11 expression changes in patient samples and TCGA database. Figure S3: quantitative analysis of western blot in Figure 3(a). Figure S4: quantitative analysis of western blot in Figure 4(d). [file 7862430.f1.zip › Figure S3 (1).pdf]

A

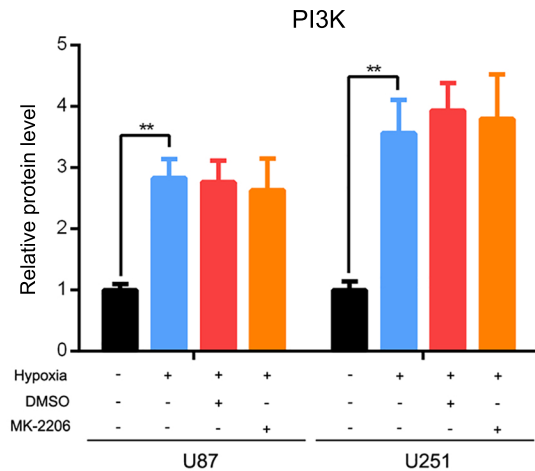

B

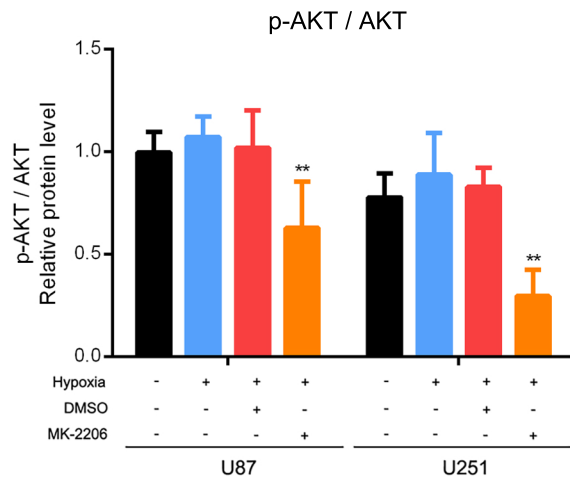

C

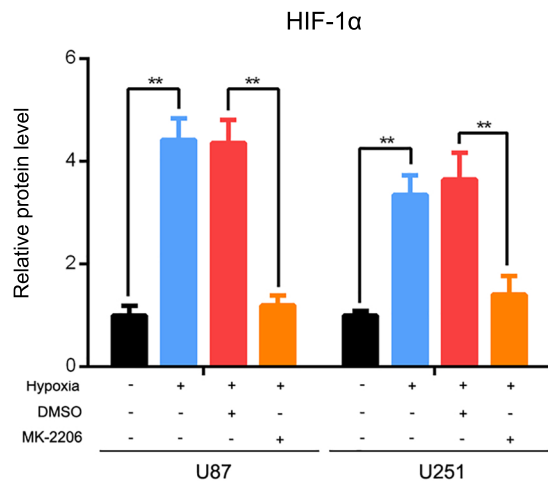

D

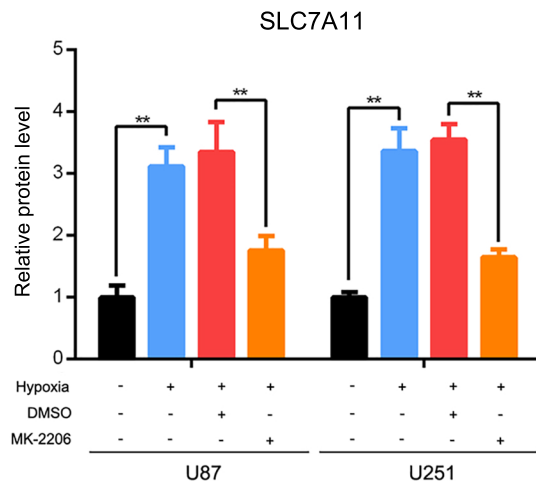

Supplement: Supplementary Materials — Table S1: primers used in this study. Figure S1: cell viability plot at the second hour and the sixth hour of hypoxic treatment. Figure S2: the SLC7A11 expression changes in patient samples and TCGA database. Figure S3: quantitative analysis of western blot in Figure 3(a). Figure S4: quantitative analysis of western blot in Figure 4(d). [file 7862430.f1.zip › Figure S4 (1).pdf]
